# MSAR: multi-stage adversarial representation learning for robust financial entity-level sentiment analysis

**DOI:** 10.3389/frai.2026.1888120

**Published:** 2026-09-11

**Authors:** Xinwen Wan, Junjie Zhao, Zheng Liang, Aoxue Shen, Wenwen Chen, Andi Xiao

**Affiliations:** School of Internet, Anhui University, Hefei, Anhui, China

**Keywords:** adaptive adversarial regularization, adversarial training, entity-level sentiment analysis, financial analysis, Model-Sensitive Metric

## Abstract

Financial entity-level sentiment analysis aims to identify sentiment polarity toward specific financial entities in financial texts. This task is challenging because a single sentence may contain multiple entities with opposite sentiment orientations, while financial sentiment is often conveyed through domain-specific and implicit cues, increasing the risk of entity confusion. To address these challenges, we propose MSAR, a Multi-Stage Adversarial Representation Learning framework built on a FinBERT-CRF backbone. At the textual level, a Model-Sensitive Metric generates adversarial examples by jointly considering model sensitivity, semantic consistency, and linguistic fluency. At the representation level, Adaptive Adversarial Regularization dynamically adjusts the regularization strength according to gradient sensitivity and output-distribution variation. By integrating discrete textual perturbation with continuous representation regularization, MSAR enhances robust entity-level sentiment prediction. Experiments on FinEntity and SentFin show that MSAR outperforms representative baselines. Ablation, sensitivity, and low-resource experiments further confirm the effectiveness and robustness of the proposed components. These findings demonstrate that jointly optimizing textual and representation-level robustness improves financial entity-level sentiment analysis, particularly under low-resource conditions.

## 1 Introduction

The rapid growth of financial textual data from news and social media has stimulated increasing interest in financial sentiment analysis. Compared with traditional document-level sentiment classification, financial entity-level sentiment analysis focuses on identifying the sentiment polarity toward specific financial entities mentioned in a text, such as companies or stocks. This task is inherently challenging because financial texts are often entity-dense and highly context-dependent. A single sentence often carries distinct or even conflicting sentiment orientations toward different entities, making it difficult to correctly identify fine-grained sentiment signals without confusion. For instance, the following statement from The Wall Street Journal—“*We see ChatGPT's prowess and traction with consumers as a near-term threat to Alphabet's multiple and a boost for Microsoft and Nvidia.”*—conveys positive sentiment toward Microsoft while implying negative implications for Alphabet. Such real-world cases highlight the strong demand for models that can handle divergent sentiment in multi-entity financial texts.

Domain-specific pre-trained language models, such as FinBERT, have achieved strong performance in financial sentiment analysis by capturing domain-specific semantics and contextual dependencies. Despite their effectiveness, these models remain highly sensitive to minor input perturbations. Small textual variations, such as synonym substitution, or slight noise injected into the embedding space, may cause large fluctuations in prediction results and even reverse sentiment judgments. In the high-stakes financial domain, such instability undermines the reliability of decision-support systems. Therefore, improving model robustness has become a crucial requirement for practical financial sentiment analysis.

To improve the robustness of pre-trained language models, adversarial training has been widely adopted in natural language processing. By introducing carefully designed perturbations during training, adversarial learning encourages models to learn smoother decision boundaries and more stable feature representations. Consequently, models become more resilient to input noise and maintain consistent prediction performance under diverse textual variations.

Nevertheless, existing adversarial learning methods still suffer from two critical drawbacks when applied to financial entity-level sentiment analysis. First, most methods only perform adversarial perturbation at a single level—either discrete textual space or continuous representation space—ignoring their complementary strengths and thus failing to defend against both types of perturbations simultaneously. Second, conventional adversarial frameworks do not explicitly model the unique characteristics of multi-entity financial texts, making it difficult for the model to separate and stabilize sentiment prediction for different targets. This often causes sentiment confusion between different entities and degrades overall classification reliability.

To address these limitations, a unified dual-level adversarial framework, termed MSAR (Multi-Stage Adversarial Representation Learning), is introduced for financial entity-level sentiment analysis. The framework performs joint perturbation learning through two hierarchical modules. The first module operates at the textual level and is referred to as MSM (Model-Sensitive Metric). Guided by an enhanced Model-Sensitive Metric scoring function, this module generates fine-grained discrete perturbations at the word and phrase levels, focusing on core financial entity features while preserving contextual coherence. The second module functions at the representation level and is defined as AAR (Adaptive Adversarial Regularization). It introduces controlled continuous perturbations to entity-specific embeddings in the latent feature space, thereby refining stable entity-level decision boundaries. Through the integration of these two modules, MSAR enables multi-scale adversarial learning with explicit entity-aware modeling. This design mitigates cross-entity sentiment conflicts, enhances model robustness and generalization ability, and preserves high classification accuracy.

The main contributions of this work are summarized as follows:

- **Construct a dual-level adversarial learning framework to enhance model robustness:** A novel MSAR framework is proposed for financial entity-level sentiment analysis by integrating discrete textual perturbation and continuous representation optimization to improve model robustness.
- **Design a model-aware perturbation generation strategy:** An MSM mechanism is introduced to identify effective adversarial perturbations by considering model sensitivity, semantic consistency, and linguistic validity.
- **Propose an adaptive robustness optimization mechanism:** An AAR strategy is developed to dynamically adjust adversarial regularization according to instance-level vulnerability, enhancing robustness while maintaining sentiment prediction capability.

## 2 Related work

Financial text sentiment analysis is an important research topic in natural language processing. Its core mission entails unearthing latent emotional indicators from multifaceted financial textual sources, including news reports, corporate disclosures, financial statements, and social media platforms. This analytical framework proves instrumental in optimizing investment decision-making, refining risk assessment protocols, and advancing market trend prognostication.

### 2.1 Financial sentiment analysis

Early studies on financial sentiment analysis were largely inspired by general opinion mining research. Hu and Liu (2004) proposed a framework for extracting opinions from text and identifying their polarity. Subsequent studies extended these techniques to financial contexts. For example, Tetlock et al. (2008) quantified linguistic features in financial news and demonstrated that textual tone reflects market sentiment and firm fundamentals. However, general-purpose sentiment lexicons often perform poorly in financial texts because many domain-specific terms have different semantic implications. To address this issue, Loughran and McDonald (2011) developed a finance-specific sentiment dictionary that clearly improved sentiment measurement in financial documents. Nevertheless, lexicon-based approaches rely heavily on manual design and struggle to capture implicit sentiment and contextual semantics in complex financial narratives.

### 2.2 Financial entity-level sentiment analysis

Financial sentiment analysis corpora have progressed from coarse-grained overall sentiment annotation to fine-grained entity-aware labeling. Early datasets, such as the Financial Phrase Bank (Malo et al., 2013), use sentences as the basic unit and annotate financial news and disclosures as positive, neutral, or negative. This provided the first domain-specific benchmark and was widely used to evaluate traditional machine learning and early deep learning models. However, each text is assumed to carry a single sentiment signal, limiting the capture of sentiment differences among multiple entities. The SEntFiN 1.0 dataset introduced entity-sensitive annotations, explicitly linking sentiment to financial entities (Sinha et al., 2022). This shifted analysis from the document level to the entity level and revealed the potential of deep semantic models for multi-entity texts. Yet, differentiated sentiment across entities within the same text was not systematically modeled. FinEntity further standardized entity spans and sentiment polarities in Reuters news (Tang et al., 2023). It explicitly accounts for cases where multiple entities exhibit distinct or even conflicting sentiment. By providing a unified benchmark for entity-level financial sentiment, FinEntity enables systematic evaluation of pre-trained models. This transition marks a critical shift from entity awareness to precise object-level sentiment modeling in financial analysis resources.

### 2.3 Pre-trained language models for financial NLP

The emergence of deep learning has advanced financial text sentiment analysis. Early neural approaches leveraged distributed word representations such as Word2Vec to capture semantic relationships in financial texts (Mikolov et al., 2013). Subsequent studies applied recurrent neural architectures, including RNN and LSTM models, to model sequential dependencies in financial news and social media data (Sohangir et al., 2018; Li et al., 2019). Although these methods improved contextual modeling compared with traditional machine learning techniques, their ability to capture long-range dependencies and complex semantic structures in financial texts remained limited. The introduction of pre-trained language models (PLMs), particularly BERT, marked a paradigm shift in natural language processing. Through large-scale pre-training, BERT learns deeply contextualized representations that improve performance across a wide range of NLP tasks. To better adapt BERT to the financial domain, Araci further pre-trained BERT on large-scale financial corpora and proposed FinBERT, which achieved substantial improvements in financial sentiment analysis tasks (Araci, 2019). Recent studies have also explored the use of large language models for financial text analysis (Kirtac and Germano, 2024). Nevertheless, existing pre-trained models still face challenges in capturing implicit sentiment signals in complex financial narratives, especially when multiple entities with heterogeneous sentiment orientations appear within the same text.

### 2.4 Adversarial training for financial sentiment analysis

**Figure 1:**
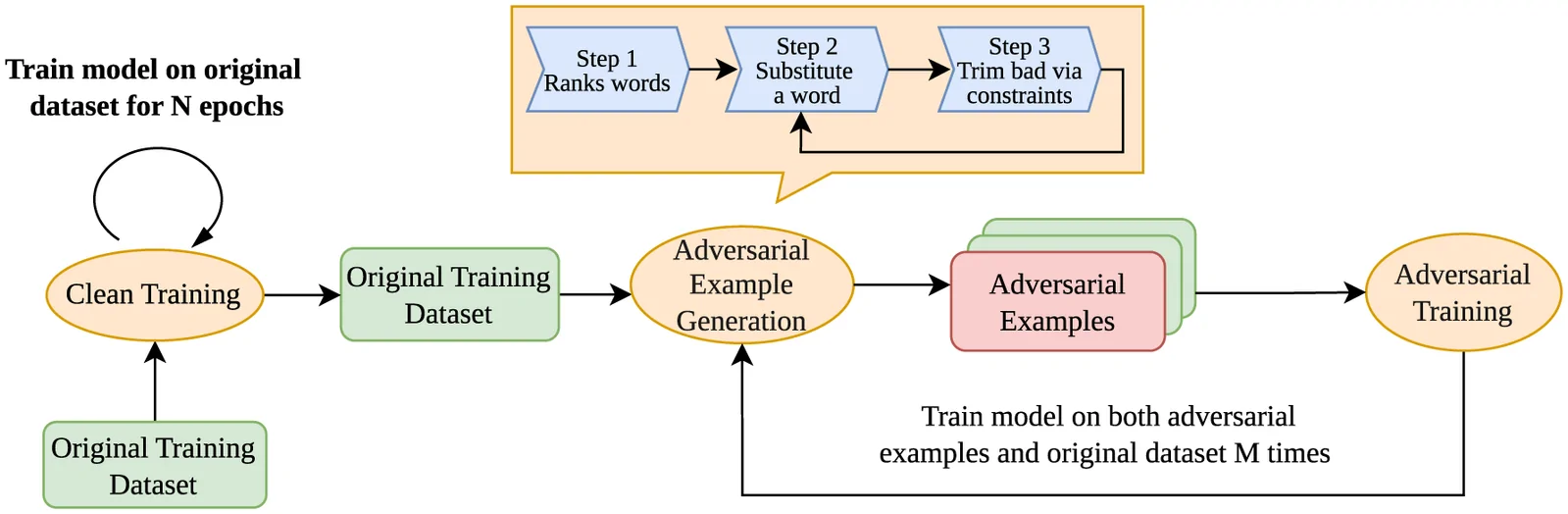
Overview of the adversarial training framework for financial sentiment analysis.

Recent years have seen robustness to semantic perturbations become an important research focus in natural language processing. Adversarial training has been widely adopted to improve model stability by introducing adversarial examples during the training process. Figure 1 illustrates the general paradigm of adversarial training, where adversarial samples are generated and incorporated into model learning to enhance robustness. According to the framework summarized by Morris, the generation of textual adversarial examples typically involves three components: a search algorithm for identifying candidate perturbations, a transformation module that modifies the original input (e.g., synonym substitution), and a set of constraints that ensures the perturbed text preserves the semantic meaning and fluency of the original sentence (Morris et al., 2020).

In the discrete text space, adversarial attacks are commonly formulated as combinatorial search problems. Since the perturbation space grows rapidly with sentence length, heuristic search strategies are typically adopted to efficiently identify adversarial modifications. Representative approaches include beam-search-based attacks (Ebrahimi et al., 2018), genetic algorithm–based methods (Alzantot et al., 2018), and greedy strategies guided by word importance scores (Gao et al., 2018; Jin et al., 2019). These methods generate adversarial samples by modifying discrete tokens while maintaining semantic consistency.

Beyond adversarial attacks in discrete text space, another line of research focuses on adversarial training in continuous representation space, where gradient-based perturbations are applied to word or embedding representations. Early methods such as FGSM (Waghela et al., 2024) introduce single-step perturbations to improve model robustness by enforcing prediction consistency within local embedding neighborhoods. Subsequent studies further explore stronger adversarial optimization strategies. For instance, FreeLB (Zhu et al., 2020) iteratively updates embedding perturbations during training to better approximate worst-case adversarial risks. Other approaches, including CreAT (Wu et al., 2023) and SeqVAT (Chen et al., 2020), enhance adversarial training by improving perturbation search or introducing multi-step optimization mechanisms.

In addition to explicit perturbation methods, several studies introduce adversarial regularization techniques to improve the robustness of pre-trained language models. SMART (Jiang et al., 2020) proposes a robust fine-tuning framework based on the proximal point method to stabilize model optimization. Similarly, R3F and R4F (Aghajanyan et al., 2020) improve generalization by introducing trust-region-based regularization during fine-tuning. In summary, although prior studies have advanced financial sentiment analysis through domain-specific datasets, pre-trained models, and adversarial learning techniques, robust entity-level sentiment modeling under multi-scale perturbations remains insufficiently explored.

## 3 Multi-stage adversarial representation learning

**Figure 2:**
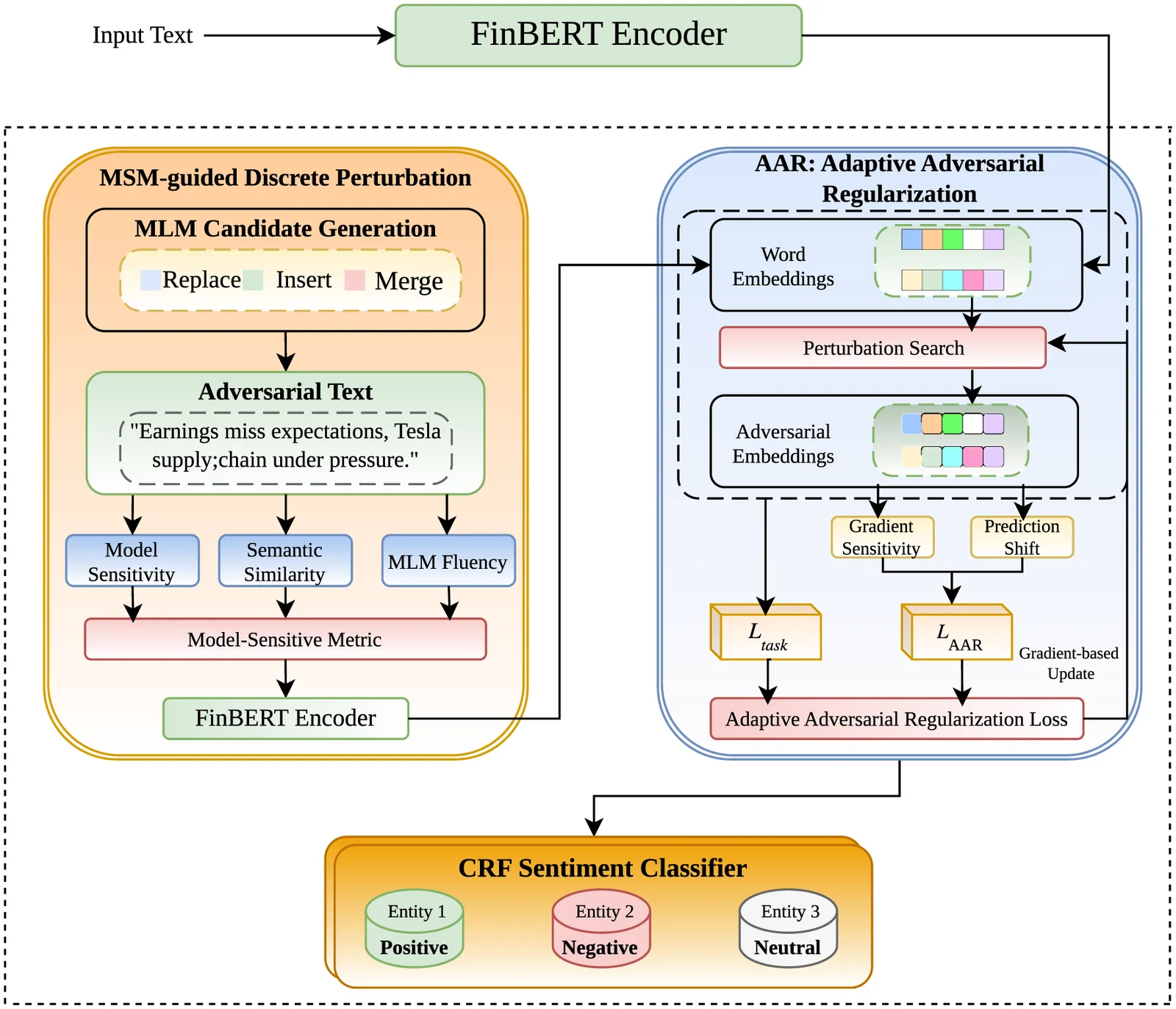
Overview of the proposed Multi-Stage Adversarial Representation Learning (MSAR) framework for robust financial entity-level sentiment analysis. FinBERT extracts entity-aware representations from input financial text, followed by dual-level adversarial optimization. The Model-Sensitive Metric (MSM) generates discrete adversarial samples via MLM and filters perturbations by prediction sensitivity, semantic consistency and fluency. The Adaptive Adversarial Regularization (AAR) applies continuous embedding perturbations with dynamically tuned regularization based on local model sensitivity. Optimized representations are then sent to the CRF decoder for consistent multi-entity sentiment predictions.

As illustrated in Figure 2, the MSAR framework consists of two adversarial levels designed to enhance the robustness of financial entity-level sentiment analysis. Given an input financial text, contextual representations are first obtained using a FinBERT encoder. The first level, referred to as the *Discrete Perturbation level*, operates in the discrete textual space. A masked language model (MLM) generates candidate perturbations through replacement, insertion, and merge operations. These candidates form adversarial texts, which are evaluated using three criteria: model sensitivity, semantic similarity, and MLM fluency. A multi-criteria selection strategy is then applied to identify effective adversarial samples while preserving semantic consistency. The selected samples are subsequently encoded by FinBERT for further processing. The second level, termed the *Continuous Perturbation level*, introduces adversarial perturbations in the continuous representation space. Gradient-based optimization is employed to add adversarial noise to the word embedding space. The perturbation search considers both gradient sensitivity and prediction variation to construct adversarial embeddings, thereby improving representation robustness and stabilizing model predictions. During training, the classification loss is jointly optimized with the proposed adaptive regularization objective under the AAR mechanism. This objective dynamically balances classification performance and robustness during model optimization. Finally, the resulting representations are fed into a CRF-based sentiment classifier, which predicts sentiment polarity (positive, negative, or neutral) for multiple financial entities within a text.

### 3.1 MSM-guided discrete perturbation

Given an input sequence *X* = {*x*_1_, *x*_2_, …, *x*_*n*_} with the corresponding ground-truth label *y*, the importance of each token for the model prediction is first estimated to identify candidate perturbation positions. Thereby, the target positions for perturbation are determined using a gradient-based saliency method, which quantifies the contribution of each token to the prediction. The input sentence is tokenized and then mapped to the embedding level of the classification model to form a sequence of word vectors: $E=\left\{{\text{e}}_{1},{\text{e}}_{2},\ldots ,{\text{e}}_{n}\right\},\;{\text{e}}_{i}\in {\mathbb{R}}^{d}$, where *d* denotes the embedding dimension. The embedded sequence is fed into the classification model to obtain the model's predicted probability *p*(*y*|*x*) for the ground truth label *y*. Based on this prediction, the cross-entropy loss function is defined in Equation 1:


$$\begin{array}{l} L_{\text{CE}}=-\log p\left(y|x\right) \end{array}$$ (1)

The gradient of the loss with respect to each token embedding is computed to quantify the influence of the token on the model prediction. The token saliency *s*_*i*_ is defined as the $L_{2}$ norm of the gradient vector in Equation 2:


$$\begin{array}{l} s_{i}=||\frac{\partial L\left(x,y\right)}{\partial {\text{e}}_{i}}{||}_{2} \end{array}$$ (2)

This score *s*_*i*_ characterizes the sensitivity of the model prediction to perturbations in the corresponding word. The words are ranked in descending order according to their obtained saliency scores. Then, adversarial perturbations are applied to the positions corresponding to the highest-ranked scores using the designed perturbation operations. Through this saliency-guided attack strategy, the perturbation process focuses on a small number of critical positions, effectively reducing the search space while maintaining strong attack effectiveness.

Having identified the critical positions for modification, the framework implements three fundamental perturbation operations: substitution, insertion and merging. Specifically, for each selected position, distinct masking strategies are applied, and the model generates replacement or additional text based on the unmasked context, thereby achieving localized modifications to the input.

**Replace**: A Replace action substitutes the token at a given position i with an alternative (e.g., changing “fantastic” to “amazing” in “The movie is fantastic.”). It first replaces *x*_*i*_ with a mask, and then selects a token *z* from a candidate set *Z* to fill in:


$$\tilde{x}=\left\{x_{1}\cdots x_{i-1}\left[\text{MASK}\right]x_{i+1}\cdots x_{n}\right\},$$

$$\text{replace}\left(x,i\right)=\left\{x_{1}\cdots x_{i-1}zx_{i+1}\cdots x_{n}\right\}.$$

For clarity, replace (*x*,*i*) is denoted by ${\tilde{x}}_{z}$. To construct an effective adversarial example, the replacement token *z* is expected to satisfy the following desiderata:

- it should be compatible with the unmasked contextual environment;
- the resulting sequence ${\tilde{x}}_{z}$ should preserve the semantics of the original input *x*;
- ${\tilde{x}}_{z}$ should induce a substantial degradation in the predictive confidence of the target model *f* on the gold label.

Unlike prior approaches that rely on hard filtering thresholds or greedy selection based on a single criterion, we introduce a unified MSM to jointly evaluate candidate perturbations. For each selected position *i*, a candidate set *Z* is constructed by querying a pre-trained MLM and retaining the Top-*k* predicted tokens:


Z={z∈V∣z∈Top-k(MLM(x,i))},

where *V* denotes the vocabulary of the MLM. For each candidate *z*∈*Z*, the MSM score for the corresponding perturbed sequence is computed using Equation 3 ${\tilde{x}}_{z}$:


$$\begin{array}{l} \mathrm{MSM}\left(x,{\tilde{x}}_{z}\right)=\frac{\Delta m\cdot \mathrm{Sim}\left(x,{\tilde{x}}_{z}\right)}{1+\max\left(0,\;\Delta \text{LM}\left(x,{\tilde{x}}_{z}\right)\right)} \end{array}$$ (3)

The model prediction sensitivity is defined in Equation 4 Δ_*m*_ measures the decrease in the model confidence assigned to the gold label after perturbation:


$$\begin{array}{l} \Delta_{m}=\max\left(0,p_{f}\left(y|x\right)-p_{f}\left(y|{\tilde{x}}_{z}\right)\right) \end{array}$$ (4)

where *p*_*f*_(*y*∣*x*) denotes the confidence assigned by the target model *f* to the true label *y* for the original input *x*, while $p_{f}\left(y|{\tilde{x}}_{z}\right)$ denotes the corresponding confidence for the perturbed input ${\tilde{x}}_{z}$. This term quantifies the extent to which the perturbation decreases the model's confidence in the correct label and therefore directly reflects the effectiveness of the adversarial perturbation. Since both confidence scores lie in [0, 1], the resulting Δ_*m*_ is also bounded within [0, 1].

Sim (*x*,$\tilde{x}$) quantifies semantic consistency. It computes the cosine similarity between sentence embeddings, ϕ(*x*) and $\phi \left({\tilde{x}}_{z}\right)$. These embeddings are frozen [CLS] token representations extracted via a pre-trained FinBERT encoder. This metric enforces preservation, ensuring adversarial fidelity to the original intent. The semantic consistency score is defined in Equation 5.


$$\begin{array}{l} \mathrm{Sim}\left(x,{\tilde{x}}_{z}\right)=\frac{1+\cos\left(\phi \left(x\right),\phi \left({\tilde{x}}_{z}\right)\right)}{2} \end{array}$$ (5)

To encourage linguistic naturalness of adversarial candidates, a fluency signal derived from a pre-trained masked language model is incorporated. Specifically, given a candidate sentence ${\tilde{x}}_{z}$, the relative log-likelihood difference with respect to the original input is computed using Equation 6:


$$\begin{array}{l} \Delta LM\left(x,{\tilde{x}}_{z}\right)=-\frac{1}{\left|{\tilde{x}}_{z}\right|}\log P_{LM}\left({\tilde{x}}_{z}\right)+\frac{1}{\left|x\right|}\log P_{LM}\left(x\right) \end{array}$$ (6)

A larger ΔLM signifies a degradation in fluency relative to the original input. The term max(0, ΔLM) selectively penalizes candidates with reduced fluency, avoiding excessive rewards for those that are more fluent. Furthermore, the log-likelihood is normalized by sequence length to mitigate length bias. The multiplicative interaction between Δ*m* and $\mathrm{Sim}\left(x,{\tilde{x}}_{z}\right)$ enforces that a candidate is considered effective only when it simultaneously induces noticeable prediction change while preserving semantic consistency. The denominator term penalizes candidates with degraded fluency, such that adversarial examples introducing substantial linguistic distortion are down-weighted during selection.

The *Insert* and *Merge* actions differ from *Replace* in terms of masking strategies. The alternative token *z* is selected analogously to that in a *Replace* action.

**Insert**: This aims to add extra information to the input (e.g., changing “I recommend...” to “I highly recommend...”). It inserts a mask after xi and then fills it. Slightly overloading the notations,


$$\tilde{x}=\left\{x_{1}\cdots x_{i}\left[\text{MASK}\right]x_{i+1}\cdots x_{n}\right\},$$

$$\text{Insert}\left(x,i\right)=\left\{x_{1}\cdots x_{i}zx_{i+1}\cdots x_{n}\right\}.$$

This increases the sequence length by 1.

**Merge**: This masks out a bigram *x*_*i*_*x*_*i*+1_ with a single mask and then fills it, reducing the sequence length by 1:


$$\tilde{x}=\left\{x_{1}\cdots x_{i-1}\left[\text{MASK}\right]x_{i+2}\cdots x_{n}\right\},$$

$$\text{Merge}\left(x,i\right)=\left\{x_{1}\cdots x_{i-1}zx_{i+2}\cdots x_{n}\right\}.$$

*z* can be the same as one of the masked tokens (e.g., masking out “New York” and then filling in “York”). This can be seen as deleting a token from the input.

**Figure 3:**
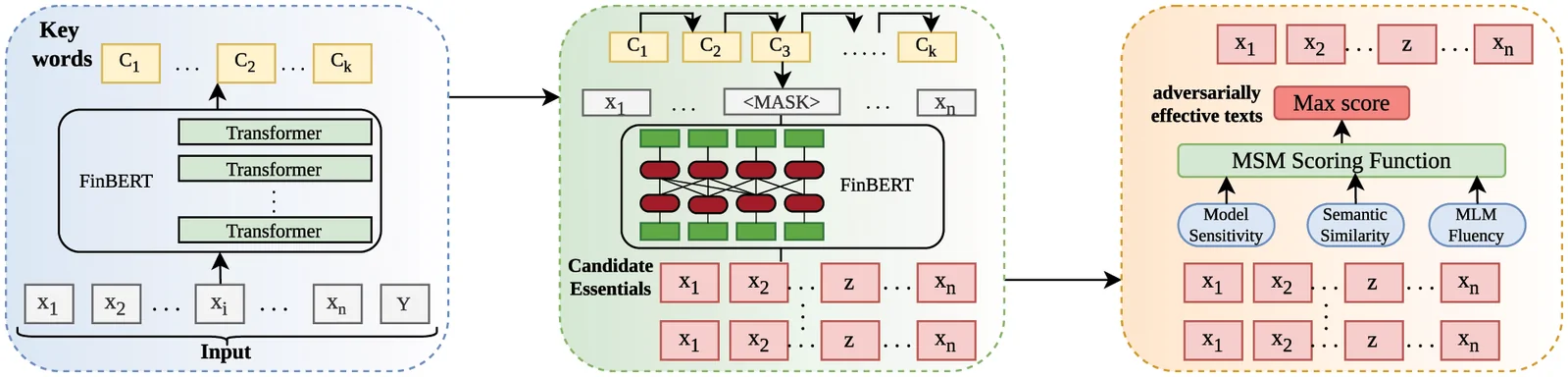
Illustration of the Model-Sensitive Metric (MSM) guided discrete adversarial perturbation generation process. The input sentence is first analyzed through gradient-based token importance estimation to identify vulnerable positions. MLM-based candidate tokens are then generated through replacement, insertion, and merging operations. Each candidate perturbation is evaluated by jointly considering model prediction sensitivity, semantic similarity, and linguistic fluency. The candidate with the highest MSM score is selected as the final adversarial sample for robustness training.

For *Insert* and *Merge*, *z* is chosen in the same manner as Replace action. In summary, for each position *i* in the input sequence *x*, the framework constructs three localized perturbation actions: replace, insert, and merge. Candidate tokens are generated by a masked language model and evaluated using the proposed MSM. The candidate with the highest MSM score is selected to construct adversarial examples. These operations allow flexible transformation of the input sequence while preserving semantic consistency. The overall MSM-guided discrete adversarial perturbation generation process is illustrated in Figure 3.

### 3.2 Adaptive adversarial regularization

Financial texts contain entities with varying sensitivity across different positions and contexts. A globally fixed regularization coefficient tends to over-constrain simple samples while under-constraining sensitive ones, leading to unstable entity-level representations. To address this issue, we propose AAR, which adaptively adjusts perturbation strength at the instance level based on gradient sensitivity and output distribution sensitivity.

Unlike the original SMART regularization framework, where the regularization coefficient is fixed across all training instances, AAR introduces an instance-adaptive mechanism. This design relaxes the implicit assumption of uniform sensitivity over the input space and output distributions, which rarely holds in practice. Specifically, the regularization strength is determined by two complementary factors: gradient sensitivity and output distribution sensitivity, enabling more precise and model-aware perturbation control.

An explicit adversarial regularization term is incorporated during fine-tuning to encourage local smoothness and improve robustness. Let *f*(·;θ) denote the FinBERT-CRF model, and let $D={\left\{\left(x_{i},y_{i}\right)\right\}}_{i=1}^{n}$ be the training dataset, where *x*_*i*_ represents the input embedding sequence obtained from the embedding layer and *y*_*i*_ denotes the corresponding token-level entity sentiment label sequence. The overall training objective is defined in Equation 7:


$$\begin{array}{l} F\left(\theta \right)=\frac{1}{n}\overset{n}{\underset{i=1}{\sum }}\left[\ell_{\text{CRF}}\left(f\left(x_{i};\theta \right),y_{i}\right)+\lambda_{s}\left(x_{i}\right)r_{s}\left(x_{i};\theta \right)\right] \end{array}$$ (7)

where ℓ_CRF_ denotes the supervised CRF loss, *r*_*s*_(*x*_*i*_; θ) is the instance-level smoothness-inducing adversarial regularization term, and λ_*s*_(*x*_*i*_) is the adaptive regularization weight for the *i*-th training sample. In contrast to conventional methods that use a fixed global coefficient λ_*s*_>0, we propose an instance-dependent smoothness weight is defined in Equation 8:


$$\begin{array}{l} \lambda_{s}\left(x_{i}\right)=\sqrt{S_{\text{grad}}\left(x_{i}\right)\cdot S_{\text{out}}\left(x_{i}\right)} \end{array}$$ (8)

In this formulation, *S*_grad_(*x*_*i*_) measures the gradient sensitivity of the supervised CRF loss with respect to the input embedding sequence, while *S*_out_(*x*_*i*_) captures the sensitivity of the token-level output distribution under small perturbations. Together, these two terms enable more precise and instance-aware control of the adversarial smoothness constraint.

Specifically, the gradient sensitivity is defined in Equation 9 *S*_grad_(*x*_*i*_) is defined as the *L*_2_ norm of the gradient of the supervised CRF loss with respect to the input embedding:


$$\begin{array}{l} S_{\text{grad}}\left(x_{i}\right)=||\nabla_{x_{i}}\ell_{\text{CRF}}\left(f\left(x_{i};\theta \right),y_{i}\right){||}_{2} \end{array}$$ (9)

which reflects how sensitive the model prediction is to small changes in the input representation.

The output sensitivity is defined in Equation 10 *S*_out_(*x*_*i*_) is defined as the KL divergence between the token-level emission distributions before and after a small perturbation:


$$\begin{array}{l} S_{\text{out}}\left(x_{i}\right)=D_{\text{KL}}\left(p_{\theta }\left(\cdot |x_{i}\right)∥p_{\theta }\left(\cdot |x_{i}+\delta \right)\right) \end{array}$$ (10)

where δ is sampled from a small norm-bounded neighborhood, and *p*_θ_(·∣*x*_*i*_) denotes the token-level emission distribution before CRF decoding. This term measures how much the output distribution changes under local perturbations. In practice, both *S*_grad_(*x*_*i*_) and *S*_out_(*x*_*i*_) are normalized within each mini-batch, and the resulting adaptive weight is clipped to [0, λ_max_] for numerical stability. To balance the contributions of the two factors, the geometric mean is adopted, which prevents either term from dominating the adaptive weight and ensures stable scaling of λ_*s*_(*x*_*i*_).

The instance-level adversarial smoothness regularizer is defined in Equation 11:


$$\begin{array}{l} r_{s}\left(x_{i};\theta \right)=\underset{||{\tilde{x}}_{i}-x_{i}{||}_{p}\leq \epsilon }{\max}\ell_{s}\left(p_{\theta }\left(\cdot |{\tilde{x}}_{i}\right),p_{\theta }\left(\cdot |x_{i}\right)\right) \end{array}$$ (11)

where ϵ>0 controls the perturbation magnitude. For the FinBERT-CRF sequence labeling task, ℓ_*s*_ is computed over the token-level emission distributions before CRF decoding and is defined as the symmetrized KL divergence is defined in Equation 12:


$$\begin{array}{l} \ell_{s}\left(P,Q\right)=D_{\text{KL}}\left(P∥Q\right)+D_{\text{KL}}\left(Q∥P\right) \end{array}$$ (12)

The inner maximization in *r*_*s*_(*x*_*i*_; θ) is approximately optimized using projected gradient ascent.

The proposed regularizer is built upon prior research on adversarial smoothness regularization. Similar formulations have been investigated for semi-supervised learning by Miyato et al. (2019) and for unsupervised domain adaptation under the ℓ_2_ constraint by Shu et al. (2018). This line of work was further extended by Zhang et al. (2019), who introduced the ℓ_∞_ norm into adversarial training for image classification. Unlike previous methods that adopt a globally fixed regularization strength, our approach incorporates an instance-adaptive weighting mechanism, which explicitly accounts for variations in local sensitivity across both the input representation space and the output distribution. This enables the regularization to dynamically concentrate on regions where the model exhibits higher vulnerability to perturbations, which is particularly important in the high-dimensional representation spaces of pre-trained language models.

**Figure 4:**
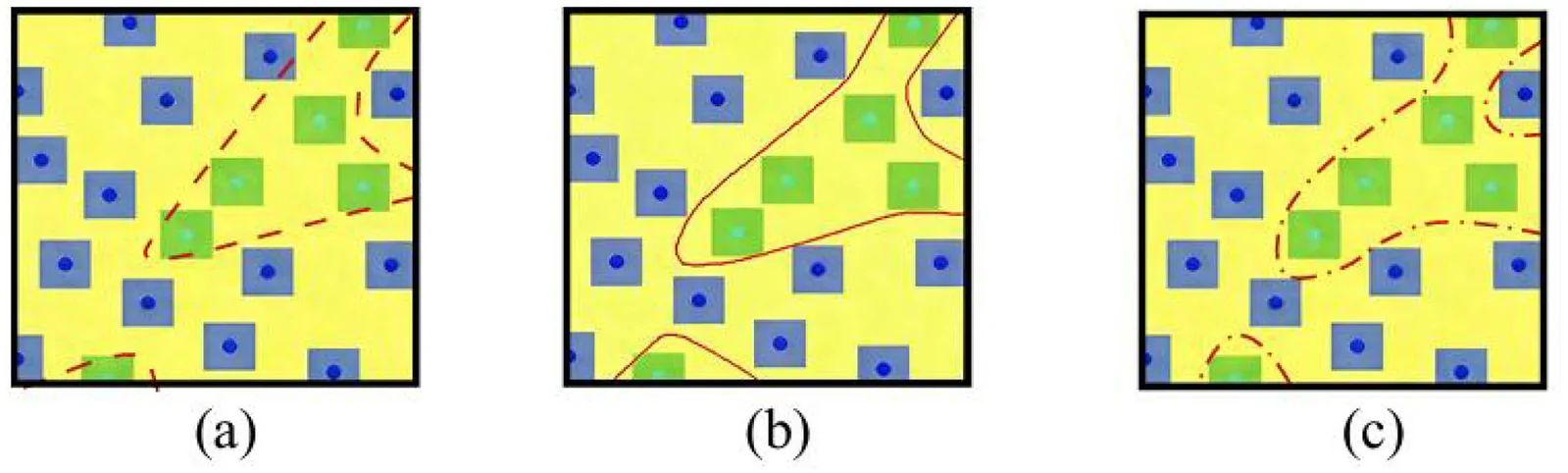
Conceptual illustration of decision boundaries learned without smoothness regularization **(a)**, with uniform smoothness-inducing adversarial regularization **(b)**, and with the proposed instance-adaptive adversarial regularization **(c)**. The red dotted curve in **(b, c)** denotes the decision boundary obtained in **(a)**. While uniform regularization enforces global smoothness, the adaptive formulation selectively strengthens regularization in locally sensitive regions, resulting in smoother yet more flexible decision boundaries around training data points.

From a theoretical perspective, the smoothness-inducing adversarial regularizer can be interpreted as encouraging local smoothness of the model *f*(·;θ) under norm-bounded perturbations. This property promotes output stability when small perturbations are applied to the input representation *x*_*i*_, encouraging smooth behavior within the local neighborhood of each training instance. In particular, the adaptive weight λ_*s*_(*x*_*i*_) serves as a data-dependent proxy for local sensitivity, rather than a fixed global coefficient. It modulates the strength of the smoothness constraint according to input-specific sensitivity. By minimizing the overall objective, the regularizer reinforces local stability while adaptively calibrating the regularization strength, thereby balancing robustness and flexibility in the learned model. An illustration of this effect is provided in Figure 4.

Such a smoothness property is particularly beneficial in low-resource settings, where limited annotated data amplify model sensitivity and increase the risk of overfitting. By imposing stronger constraints on locally unstable regions, the proposed regularization improves generalization performance, especially when only a small proportion of labeled training data is available. This observation is further supported by the low-resource experiments reported in Section 4.6. Finally, the idea of measuring local smoothness is closely related to the notion of local shift sensitivity in robust statistics, which describes how model outputs respond to small input perturbations and aligns with the proposed formulation of local sensitivity-aware regularization (Hampel, 1974; Huber, 2011). This formulation can therefore be viewed as a modern instantiation of this principle in neural model fine-tuning.

### 3.3 CRF-based sentiment inference method

**Figure 5:**
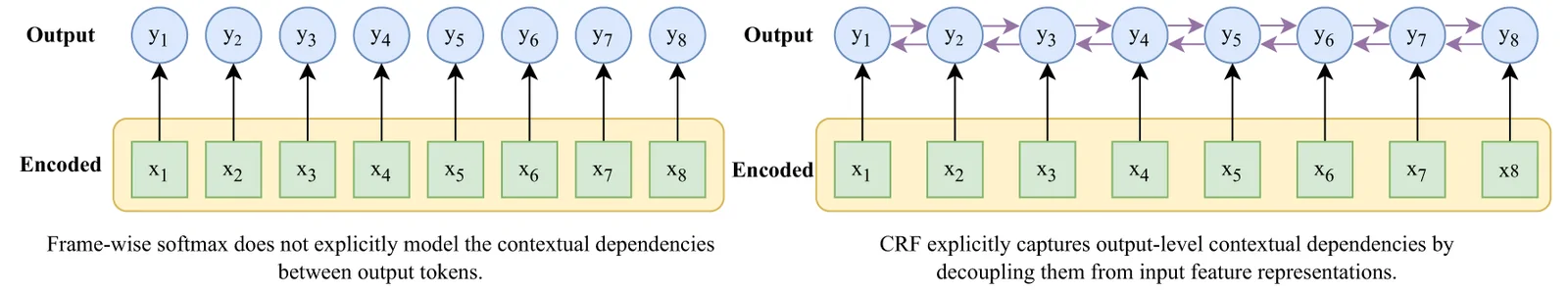
Comparison between frame-wise token classification and CRF-based structured inference. **Left**: token-level softmax predicts each label independently without modeling output dependencies. **Right**: CRF explicitly captures inter-token label dependencies while decoupling them from input feature representations.

Financial entity-level sentiment analysis is essentially a sequence labeling task, where label dependencies exist across context. Sentiment polarities toward different entities in the same text do not change randomly, so it is necessary to model contextual transition consistency. Following the feature encoding stage, structured dependencies among output labels are further modeled using a Conditional Random Field (CRF). Figure 5 illustrates the difference between frame-wise token classification and CRF-based structured inference. While conventional token-level softmax performs independent predictions at each position, it fails to explicitly capture contextual dependencies between adjacent output tokens, which are often crucial for accurate sentiment inference (Lafferty et al., 2001).

To address this limitation, a linear-chain CRF level is applied on top of the contextualized token representations produced by the encoder. Given an input sequence *x* = (*x*_1_, …, *x*_*n*_) and the corresponding encoded features *h* = (*h*_1_, …, *h*_*n*_), the CRF global score for a label sequence is defined in Equation 13 *y* = (*y*_1_, …, *y*_*n*_) as:


$$\begin{array}{l} s\left(x,y\right)=\overset{n}{\underset{i=1}{\sum }}\psi_{\text{emit}}\left(y_{i},h_{i}\right)+\overset{n-1}{\underset{i=1}{\sum }}\psi_{\text{trans}}\left(y_{i},y_{i+1}\right) \end{array}$$ (13)

where ψ_emit_(*y*_*i*_, *h*_*i*_) denotes the emission score derived from the encoder output at position *i*, and ψ_trans_(*y*_*i*_, *y*_*i*+1_) models the transition compatibility between adjacent sentiment labels.

Unlike token-wise softmax inference, the CRF explicitly models output-level dependencies while decoupling them from the input feature representations. This design enables globally consistent sentiment predictions across the entire sequence. During training, the CRF parameters are optimized by maximizing the conditional log-likelihood of the gold label sequences. At inference time, the optimal label sequence is efficiently decoded using the Viterbi algorithm (Huang et al., 2015).

By integrating CRF-based structured inference with the proposed encoder, the proposed framework benefits from both locally stable representations and globally coherent sentiment predictions.

## 4 Experiments

### 4.1 Experimental setup

Experiments are conducted on two entity-level financial sentiment analysis datasets, **FinEntity** and **SentFin**. Both datasets are designed to evaluate sentiment polarity toward target financial entities in financial texts. FinEntity contains more diverse and complex financial contexts with imbalanced sentiment distributions, whereas SentFin consists of relatively shorter texts with more explicit sentiment expressions.

All experiments are implemented in PyTorch and conducted on a single NVIDIA RTX 4060 GPU. FinBERT is used as the backbone encoder, followed by a CRF layer for structured entity-level sentiment prediction. Each dataset is randomly split into training and validation sets with a ratio of 80% and 20%, respectively. The sentiment labels include **Positive**, **Neutral**, and **Negative**.

Macro-averaged precision, recall, and F1-score are adopted as the main evaluation metrics. Since the datasets contain imbalanced sentiment distributions, macro-averaged metrics are more suitable than accuracy because they assign equal importance to each sentiment category. Specifically, precision and recall are first computed for each category, and the F1-score is calculated as their harmonic mean. Macro-F1 is then obtained by averaging the F1-scores across the three sentiment categories.

The model is trained for 20 epochs with a batch size of 16 using the AdamW optimizer. The learning rates of the FinBERT encoder and the CRF layer are set to 3 × 10^−5^ and 1 × 10^−2^, respectively. Weight decay is set to 0.01, and the warm-up ratio is set to 0.01. Gradient clipping and a linear learning-rate scheduler are applied during training.

For MSAR, the perturbation radius ϵ and finite-difference scale ξ are both set to 1 × 10^−3^. One-step power iteration is used to generate adversarial perturbations. The adaptive regularization coefficient is computed dynamically for each sample, with the maximum value set to 1.0. To evaluate the stability and reproducibility of the proposed method, all experiments are repeated three times using different random seeds, namely 47, 48, and 49.

### 4.2 Baseline models

To comprehensively evaluate the effectiveness of the proposed MSAR framework, we compare it with representative baselines from three categories: traditional neural models, financial-domain pre-trained language models (PLMs), and large language models (LLMs).

Traditional baselines include RNN and LSTM, which represent classical sequence modeling architectures for sentiment analysis. Financial-domain PLM baselines include FinBERT-Softmax, FinBERT-CRF, RoBERTa-Finance, and Modern-FinBERT. Among them, FinBERT-Softmax uses FinBERT as the encoder and applies an independent classification layer for sentiment prediction, while FinBERT-CRF further incorporates a CRF layer to model label dependencies in entity-level sentiment prediction. RoBERTa-Finance and Modern-FinBERT (Warner et al., 2025) are included as more recent financial-domain pretrained models, allowing us to compare MSAR with stronger domain-specific PLM baselines.

Furthermore, we include LLM-based reference baselines to reflect recent progress in financial NLP. Specifically, GPT-3, Qwen, and FinGPT (Yang et al., 2023) are evaluated under a 5-shot prompting setting on the same validation split. These models are not fine-tuned on the training data; instead, they are used to assess the generalization ability of large language models under limited task-specific supervision. Since most LLMs are designed for general instruction following rather than entity-level sequence labeling, their results should be interpreted as reference performance rather than directly comparable fine-tuned sequence-labeling baselines.

These baselines provide a comprehensive comparison covering traditional neural architectures, financial-domain pre-trained encoders, and recent LLM-based financial NLP methods. Traditional and PLM-based baselines are evaluated under supervised training or fine-tuning settings, while LLM-based baselines are evaluated under a 5-shot prompting setting. This comparison allows us to examine whether the proposed MSAR framework can improve entity-level financial sentiment prediction beyond conventional sequence models, strong financial pre-trained encoders, and recent LLM-based reference methods.

### 4.3 Main results

Table 1 presents the performance comparison on the FinEntity and SentFin datasets. Overall, pre-trained language models consistently outperform traditional sequence models, demonstrating the advantage of contextualized representations for entity-level financial sentiment analysis. Among the supervised or fine-tuned baselines, financial-domain encoders such as FinBERT, RoBERTa-Finance, and Modern-FinBERT achieve stronger performance than traditional neural architectures, indicating the importance of domain-specific representation learning.

**Table 1:** Performance comparison of the proposed MSAR model and representative baseline models on the FinEntity and SentFin datasets.

Model	FinEntity			SentFin		
	Precision	Recall	Macro-F1	Precision	Recall	Macro-F1
Traditional models						
RNN	70.6 ± 0.4	69.8 ± 0.5	69.9 ± 0.5	60.8 ± 0.6	59.6 ± 0.5	59.9 ± 0.6
LSTM	75.2 ± 0.5	73.7 ± 0.6	74.6 ± 0.5	65.4 ± 0.5	64.2 ± 0.6	64.6 ± 0.5
Pre-trained models						
FinBERT-Softmax	82.4 ± 0.4	81.2 ± 0.5	81.5 ± 0.4	82.6 ± 0.4	81.8 ± 0.5	82.0 ± 0.4
FinBERT-CRF	85.2 ± 0.5	85.4 ± 0.4	84.6 ± 0.5	86.4 ± 0.4	85.1 ± 0.5	85.5 ± 0.4
RoBERTa-Finance	89.1 ± 0.4	87.3 ± 0.5	88.1 ± 0.4	88.3 ± 0.4	87.6 ± 0.5	87.8 ± 0.4
Modern-FinBERT	88.8 ± 0.5	86.2 ± 0.6	86.6 ± 0.5	87.2 ± 0.4	86.5 ± 0.5	86.7 ± 0.4
Large language models (few-shot)						
GPT-3	62.3 ± 0.7	56.8 ± 0.8	59.4 ± 0.7	68.7 ± 0.6	68.4 ± 0.6	68.4 ± 0.6
Qwen	61.1 ± 0.7	57.5 ± 0.8	58.5 ± 0.7	74.4 ± 0.6	74.2 ± 0.5	74.1 ± 0.6
FinGPT	74.8 ± 0.5	72.6 ± 0.6	73.4 ± 0.5	79.5 ± 0.5	78.8 ± 0.5	79.0 ± 0.5
**FinBERT-MSAR-CRF**	**92.5 ± 0.3**	**93.4 ± 0.4**	**92.8 ± 0.3**	**91.5 ± 0.3**	**93.0 ± 0.4**	**92.7 ± 0.3**

The table reports mean macro-averaged precision, recall, and F1 scores (%) over three independent runs. Standard deviations are reported after the ± symbol. The best results are highlighted in bold. Values are reported as mean ± standard deviation over three independent runs. All metrics are macro-averaged and expressed as percentages. The best results are highlighted in bold.

Compared with all baseline groups, the proposed MSAR achieves the best overall performance on both datasets. On the more challenging FinEntity dataset, MSAR obtains the highest macro-averaged precision, recall, and F1 scores, showing clear improvements over the strong FinBERT-CRF baseline. Similar performance gains are also observed on SentFin, suggesting that the proposed framework can generalize across different financial text distributions and sentiment expression patterns.

The LLM-based methods, including GPT-3, Qwen, and FinGPT, are evaluated as 5-shot reference baselines. Although these models show certain generalization ability without task-specific fine-tuning, their performance remains limited for entity-level sequence labeling compared with fine-tuned financial-domain encoders. This indicates that direct prompting with general or financial LLMs may still be insufficient for fine-grained entity-level sentiment prediction, where accurate entity boundary recognition and target-specific sentiment discrimination are required.

The performance improvements of MSAR mainly come from the complementary effects of MSM and AAR. The MSM module generates semantically consistent and model-sensitive perturbations, which provide more informative adversarial samples for robustness training. Meanwhile, the AAR module dynamically adjusts the regularization strength according to gradient sensitivity and output-distribution variation, preventing over-regularization on stable samples while imposing stronger smoothness constraints on vulnerable ones. By combining textual-level adversarial sample generation with representation-level adaptive regularization, MSAR learns more robust entity-aware representations and maintains more stable predictions under perturbations.

### 4.4 Ablation study

To examine the individual contributions of different components in the proposed MSAR framework, we conduct a component-wise ablation study on the FinEntity dataset. As shown in Table 2, MSM and AAR are added separately and jointly to the FinBERT-CRF backbone, while BERT-CRF and FinBERT-CRF are used as baseline models. The comparison between BERT-CRF and FinBERT-CRF shows that domain-specific pre-training is beneficial for entity-level financial sentiment analysis. Compared with the general BERT encoder, FinBERT is more suitable for capturing financial expressions, domain-specific sentiment cues, and contextual semantics. Therefore, FinBERT-CRF provides a stronger backbone for subsequent adversarial enhancement.

**Table 2:** Component-wise ablation study of the proposed MSAR framework on the FinEntity dataset.

Model	MSM	AAR	Class-wise F1			Overall performance		
			Neg	Neu	Pos	Macro-P	Macro-R	Macro-F1
BERT-CRF	–	–	75.2	76.3	77.1	76.4	77.4	76.2
FinBERT-CRF	–	–	84.2	84.4	85.2	85.2	85.4	84.6
FinBERT-CRF + MSM	Yes	–	90.1	92.8	89.3	91.0	91.0	90.7
FinBERT-CRF + AAR	–	Yes	86.2	84.1	89.7	87.0	86.5	86.7
**MSAR (ours)**	Yes	Yes	**92.3**	**93.4**	**92.6**	**92.5**	**93.4**	**92.8**

The table reports class-wise F1 scores for negative, neutral, and positive sentiment, as well as overall macro-averaged precision, recall, and F1 scores. The best result in each column is highlighted in bold.

After introducing the MSM module, the model achieves clear improvements in both class-wise and overall performance. This demonstrates that MSM is an important component in the proposed framework. By selecting model-sensitive positions for masking and substitution, MSM can generate more informative discrete perturbations than random or fixed perturbation strategies. These perturbations expose vulnerable regions of the model decision space and encourage the model to learn more discriminative sentiment representations. This is particularly useful for financial texts, where sentiment polarity is often implicit, context-dependent, or expressed through domain-specific terms. The AAR module also contributes to model robustness, but its effect is more moderate when used alone. Unlike MSM, which focuses on perturbation construction, AAR mainly improves the learning process by imposing adaptive smoothness constraints on the model representations. It encourages the model to produce stable predictions under small adversarial perturbations, thereby reducing excessive sensitivity to local input variations. However, when AAR is used without MSM, the perturbation signals may not be sufficiently aligned with sentiment-sensitive tokens, which limits its independent effectiveness. When MSM and AAR are combined, the full MSAR model obtains the best overall performance. This result indicates that the two components are complementary rather than redundant. MSM provides high-quality sentiment-sensitive perturbations, while AAR further regularizes the model to maintain prediction consistency under these perturbations. In this way, MSM enhances the effectiveness of adversarial sample construction, and AAR strengthens the stability of representation learning. The ablation results therefore verify the necessity of both components and demonstrate that their joint use leads to better robustness and generalization for entity-level financial sentiment analysis.

### 4.5 Parameter sensitivity and computational cost analysis of MSM

To further analyze the effectiveness and computational cost of the MSM module, a sensitivity analysis is conducted on the candidate search width Top-*k*. In MSM, Top-*k* determines the number of candidate substitutions retained for each selected perturbation position. A larger Top-*k* expands the candidate search space and may improve the quality of adversarial samples, but it also increases the computational cost because more candidates need to be evaluated through semantic similarity computation and classifier-based scoring. In this experiment, all hyperparameters except Top-*k* are kept fixed, and the same subset of the FinEntity dataset is used across all settings to ensure a fair comparison. Attack success rate (ASR), confidence drop, entity preservation rate, and total runtime are reported. ASR and confidence drop reflect the effectiveness of generated adversarial samples, while entity preservation rate measures whether the target financial entities remain unchanged after perturbation. Runtime records the total time required for MSM-based adversarial sample generation.

As shown in Table 3, increasing Top-*k* generally improves the adversarial effectiveness of MSM. Both ASR and confidence drop increase as Top-*k* becomes larger, indicating that a wider candidate search space helps MSM identify more effective perturbation candidates. Specifically, ASR increases from 19.00% at Top-*k* = 1 to 35.40% at Top-*k* = 20, while confidence drop increases from 0.1350 to 0.2495. These results confirm the importance of candidate search width in model-sensitive perturbation generation.

**Table 3:** Sensitivity analysis of Top-*k* in the MSM module on the FinEntity dataset.

Top-*k*	ASR (%)	Conf. drop	Entity pres. (%)	Time
1	19.00	0.1350	93.20	2h32m
3	24.80	0.1753	93.60	5h17m
5	27.20	0.1945	93.60	7h57m
10	31.00	0.2183	94.00	13h57m
15	34.00	0.2390	93.60	20h12m
20	35.40	0.2495	93.00	25h32m

The table reports the attack success rate (ASR), confidence drop, entity preservation rate, and total adversarial sample generation time under different Top-*k* settings.

However, the improvement in adversarial effectiveness is accompanied by a substantial increase in computational cost. As Top-*k* increases, the runtime grows rapidly because each additional candidate requires extra semantic similarity calculation and classifier forward evaluation. Compared with Top-*k* = 10, Top-*k* = 20 improves ASR by 4.40 percentage points, but increases the runtime from 13h57m to 25h32m. In addition, the entity preservation rate decreases from 94.00% to 93.00%, suggesting that an overly large candidate space may introduce more aggressive substitutions and slightly increase the difficulty of entity preservation.

Therefore, Top-*k* = 10 is selected as the default setting for the MSM module. This setting achieves strong adversarial generation performance while maintaining stable entity preservation and relatively lower computational cost compared with larger Top-*k* values. Overall, the results demonstrate that MSM involves a clear trade-off between perturbation effectiveness and computational efficiency, and the main computational overhead comes from the candidate evaluation stage rather than from additional model parameters.

### 4.6 Low-resource experiment

Following the experimental setup described in Section 4.1, we conduct low-resource experiments on the FinEntity dataset using 5%, 10%, 30%, 50%, 70%, and 100% of the available training data. Stratified sampling is applied to approximately preserve the original sentiment-label distribution at each data ratio. For each ratio and random seed, Base, FreeLB, SMART, and MSAR are trained on exactly the same sampled subset to ensure a fair comparison. All other settings, including data splitting, preprocessing, model configuration, training procedure, random seeds, and evaluation protocol, remain unchanged. No additional labeled or task-specific external data are introduced.

The 5% and 10% ratios represent severely resource-constrained conditions, the 30%–70% ratios represent intermediate-resource conditions, and the 100% ratio serves as the full-resource reference. Specifically, this experiment investigates: (1) how rapidly performance deteriorates as labeled data become scarce; (2) whether MSAR provides greater improvements under severe data constraints than under the full-resource setting; and (3) whether its advantages remain consistent across sentiment categories and training-data scales.

**Figure 6:**
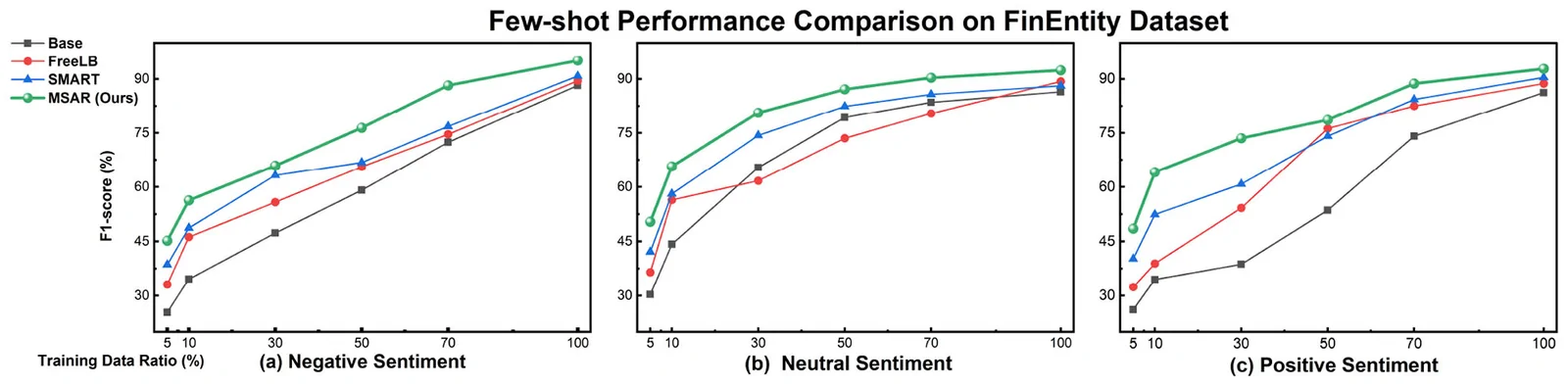
Performance comparison of different adversarial training methods under varying training data ratios on the financial sentiment analysis task. The x-axis represents the proportion of training data used for model training (5%, 10%, 30%, 50%, 70%, and 100%), while the y-axis denotes the F1-score (%). Results are reported for three sentiment categories: Negative, Neutral, and Positive. The proposed method consistently outperforms the baseline models across all data ratios, particularly in low-resource scenarios. **(a)** Negative statement. **(b)** Neutral statement. **(c)** Positive statement.

The results are reported in Table 4 and Figure 6. As expected, the performance of all methods improves as more labeled training data become available. Nevertheless, MSAR consistently achieves the highest class-wise F1 scores across all data ratios and sentiment categories, indicating that its advantage is not limited to a particular class or resource setting.

**Table 4:** Low-resource performance on the FinEntity dataset under different training-data ratios.

Ratio	Negative				Neutral				Positive			
	Base	FreeLB	SMART	MSAR	Base	FreeLB	SMART	MSAR	Base	FreeLB	SMART	MSAR
5%	42.6	48.3	51.7	**57.4**	46.8	52.1	55.6	**61.3**	40.9	47.5	50.8	**58.6**
10%	54.3	60.5	64.2	**70.1**	58.7	64.8	68.5	**74.6**	52.4	59.6	63.1	**71.2**
30%	68.5	73.2	76.6	**81.4**	72.4	76.1	79.5	**84.3**	66.7	72.8	76.4	**82.1**
50%	75.9	79.6	82.4	**86.8**	78.5	81.7	84.6	**88.5**	74.8	79.2	82.1	**87.3**
70%	80.8	84.1	86.5	**90.2**	82.6	85.3	87.8	**91.2**	80.1	84.4	86.7	**90.5**
100%	84.2	88.3	90.1	**92.3**	84.4	88.5	90.6	**93.4**	85.2	88.7	90.4	**92.6**

The table reports class-wise F1 scores (%) averaged over three runs for the negative, neutral, and positive sentiment categories. The best result in each column is highlighted in bold.

The benefits of MSAR are especially evident under severe data scarcity. With only 5% of the training data, MSAR outperforms SMART by 5.7, 5.7, and 7.8 F1 points for the negative, neutral, and positive categories, respectively. At the 10% ratio, the corresponding improvements are 5.9, 6.1, and 8.1 points. Averaged across the three sentiment categories, MSAR improves upon SMART by 6.4 points at the 5% ratio and 6.7 points at the 10% ratio. In comparison, the average improvement is reduced to 2.4 points when the complete training set is used. This trend provides direct evidence that MSAR offers greater relative benefits under low-resource conditions.

To further quantify performance degradation, we compare each method under the 5% setting with its corresponding full-resource result. When the training data are reduced from 100% to 5%, the average class-wise F1 score of MSAR decreases by 33.7 points, whereas SMART, FreeLB, and Base decrease by 37.7, 39.2, and 41.2 points, respectively. Therefore, although all methods experience substantial performance degradation under severe data scarcity, MSAR retains a larger proportion of its full-resource performance. Nevertheless, the remaining 33.7-point reduction also indicates that MSAR mitigates, but does not completely eliminate, the adverse effects of insufficient supervision.

The results also reveal where the improvements occur. The positive category obtains the largest gain over SMART in the two most resource-constrained settings, reaching 7.8 points at 5% and 8.1 points at 10%. More generally, the larger gains observed at lower data ratios suggest that conventional fine-tuning and standard adversarial training are more vulnerable to sparse supervision. Under limited supervision, models may rely more heavily on unstable lexical correlations and form insufficiently constrained decision boundaries. The observed performance pattern is consistent with the intended roles of the two MSAR components: model-sensitive perturbation exposes locally vulnerable representations, while adaptive adversarial regularization applies stronger consistency constraints to vulnerable instances. Together, these mechanisms provide a plausible explanation for the stronger performance retention of MSAR under low-resource conditions.

As the training ratio increases, the performance gap between MSAR and SMART gradually decreases, from an average of 6.4–6.7 points at the 5%–10% ratios to 2.4 points at the 100% ratio. This narrowing gap is consistent with the interpretation that additional labeled examples partially alleviate the representation instability caused by limited supervision. However, MSAR continues to outperform the competing methods even in the full-resource setting, showing that the proposed mechanisms remain beneficial beyond severe data scarcity.

Overall, these findings demonstrate that MSAR improves both data efficiency and performance retention in low-resource financial entity-level sentiment analysis. Its main advantage lies in reducing the performance degradation caused by insufficient supervision, particularly when only 5%–10% of the labeled training data are available.

## 5 Discussion

The results of this study suggest that robustness is not merely an auxiliary objective for financial entity-level sentiment analysis, but a key factor in improving fine-grained sentiment understanding. Unlike sentence-level sentiment classification, entity-level financial sentiment analysis requires the model to distinguish sentiment signals associated with different entities within the same text. This is particularly challenging when multiple entities appear in one sentence, when sentiment expressions are implicit, or when opposite sentiment orientations coexist. In such cases, prediction errors often arise not only from insufficient semantic understanding, but also from unstable entity-specific representations. Therefore, improving robustness at both the textual and representation levels is essential for reducing cross-entity sentiment confusion.

The effectiveness of MSAR can be interpreted from its dual-level robustness design. The MSM module does not simply increase the amount of training data through random perturbations. Instead, it constructs adversarial samples around model-sensitive positions, where minor textual changes are more likely to affect entity-level sentiment decisions. This encourages the model to focus on more reliable financial sentiment cues rather than superficial lexical patterns. Such a mechanism is especially important in financial texts, where sentiment is often expressed through professional terms, market expectations, comparative descriptions, or implicit event implications.

The AAR module provides complementary robustness by regularizing the local geometry of the representation space. A fixed regularization strength assumes that all samples have similar sensitivity to perturbations, which is unrealistic for financial entity-level sentiment analysis. Some samples contain explicit sentiment expressions and are relatively stable, whereas others involve ambiguous wording, multiple entities, or conflicting sentiment orientations. By assigning instance-adaptive regularization weights, AAR applies stronger constraints to locally unstable samples while avoiding unnecessary over-smoothing on easier ones. This mechanism helps preserve model flexibility while improving prediction stability.

These findings also provide useful implications for low-resource financial NLP. When labeled data are limited, models are more likely to rely on spurious correlations and unstable decision boundaries. The combination of MSM and AAR alleviates this problem from two perspectives: MSM enriches the training distribution with informative adversarial examples, while AAR constrains the model to behave smoothly around sensitive representations. This explains why the proposed framework remains effective when the amount of annotated data is reduced. More broadly, robustness-oriented learning can serve as a practical strategy for improving generalization in specialized financial NLP tasks where large-scale labeled data are difficult to obtain.

Overall, MSAR demonstrates that combining model-sensitive textual perturbation with adaptive representation regularization is a promising direction for robust financial entity-level sentiment analysis. The contribution of the framework is not limited to improving classification performance; more importantly, it encourages the model to learn stable, entity-aware, and perturbation-resistant sentiment representations. This provides a useful foundation for building more reliable financial NLP systems in scenarios involving multi-entity texts, implicit sentiment expressions, and limited annotated data.

From the perspective of foundation model adaptation, this study also provides insights into robust fine-tuning of domain-specific pretrained models. Although large-scale pre-training enables foundation models to acquire broad linguistic and semantic knowledge, their downstream performance still highly depends on the fine-tuning strategy adopted for specific tasks and domains. In financial applications, limited annotated data, domain-specific terminology, and distribution variations may cause pretrained models to become sensitive to local perturbations during adaptation. The proposed MSAR framework addresses this issue by introducing robustness-aware fine-tuning mechanisms at both the input and representation levels. MSM improves the adaptation process by generating informative adversarial examples that expand the effective training distribution, while AAR dynamically regulates representation sensitivity according to sample-level vulnerability. Therefore, this work highlights that effective foundation model adaptation requires not only domain-specific pre-training but also robust optimization strategies during downstream fine-tuning. The proposed framework provides a potential paradigm for improving the reliability of financial foundation models and can be further extended to other domain-specific NLP applications.

## 6 Limitations and future work

Despite these advantages, MSAR still has several limitations. First, the textual-level perturbation process introduces additional computational cost because candidate generation, semantic similarity evaluation, fluency estimation, and model-sensitive scoring are required. Although a moderate Top-*k* value can balance adversarial effectiveness and efficiency, the computational overhead may still limit the application of MSAR to larger datasets or real-time systems. Second, the current experiments mainly focus on financial entity-level sentiment analysis. Whether MSAR can generalize to other financial NLP tasks, such as event extraction, risk classification, relation extraction, and financial question answering, still requires further investigation. Third, the current perturbation strategy mainly relies on masked language model candidates and similarity-based constraints, which may limit the diversity of adversarial examples to local lexical or phrase-level variations.

Future work can be extended in several directions. First, more efficient candidate filtering and scoring strategies can be developed to reduce the computational cost of MSM. For example, lightweight candidate pre-screening or approximate scoring mechanisms may help improve efficiency without substantially weakening adversarial sample quality. Second, the adaptive regularization mechanism can be further refined at the entity level. Instead of assigning one adaptive weight to the whole input instance, future models can assign different robustness constraints to different entities within the same text, which may better handle multi-entity sentences with conflicting sentiment orientations. Third, future research can explore more diverse perturbation strategies beyond token substitution, such as event-aware perturbation, entity-relation-aware perturbation, or discourse-level rewriting. These strategies may better reflect real financial text variations caused by market events, reporting styles, or changes in professional terminology. Finally, integrating MSAR with stronger financial large language models may provide more context-aware adversarial examples and improve interpretability, especially in complex financial scenarios that require reasoning over entities, events, and implicit sentiment cues.

## Data Availability

The original contributions presented in the study are included in the article/supplementary material, further inquiries can be directed to the corresponding author.
